# Functional connectivity in mild cognitive impairment with Lewy bodies

**DOI:** 10.1007/s00415-021-10580-z

**Published:** 2021-04-29

**Authors:** Julia Schumacher, John-Paul Taylor, Calum A. Hamilton, Michael Firbank, Paul C. Donaghy, Gemma Roberts, Louise Allan, Rory Durcan, Nicola Barnett, John T. O’Brien, Alan J. Thomas

**Affiliations:** 1 Translational and Clinical Research Institute, Faculty of Medical Sciences, Newcastle University, Campus for Ageing and Vitality, Newcastle upon Tyne, NE4 5PL UK; 2 Institute of Health Research, University of Exeter, Exeter, UK; 3 Department of Psychiatry, University of Cambridge School of Medicine, Cambridge, CB2 0SP UK

**Keywords:** Resting-state fMRI, Dynamic connectivity, Sliding-window analysis, Leading eigenvector dynamic analysis, Lewy body dementia, Alzheimer’s disease

## Abstract

Previous resting-state fMRI studies in dementia with Lewy bodies have described changes in functional connectivity in networks related to cognition, motor function, and attention as well as alterations in connectivity dynamics. However, whether these changes occur early in the course of the disease and are already evident at the stage of mild cognitive impairment is not clear. We studied resting-state fMRI data from 31 patients with mild cognitive impairment with Lewy bodies compared to 28 patients with mild cognitive impairment due to Alzheimer’s disease and 24 age-matched controls. We compared the groups with respect to within- and between-network functional connectivity. Additionally, we applied two different approaches to study dynamic functional connectivity (sliding-window analysis and leading eigenvector dynamic analysis). We did not find any significant changes in the mild cognitive impairment groups compared to controls and no differences between the two mild cognitive impairment groups, using static as well as dynamic connectivity measures. While patients with mild cognitive impairment with Lewy bodies already show clear functional abnormalities on EEG measures, the fMRI analyses presented here do not appear to be sensitive enough to detect such early and subtle changes in brain function in these patients.

**Supplementary Information:**

The online version contains supplementary material available at 10.1007/s00415-021-10580-z.

## Introduction

Dementia with Lewy bodies (DLB) is the second most common form of neurodegenerative dementia after Alzheimer’s disease (AD) [1, 2]. Similar to AD, the dementia stage is often preceded by a period in which cognitive decline is already present, but independence in activities of daily living is still preserved [3, 4]. This is referred to as mild cognitive impairment with Lewy bodies (MCI-LB). Patients with DLB and MCI-LB often show transient changes in cognition such as fluctuations in attention, arousal, and alertness that mostly occur spontaneously without any situational explanation [5, 6]. Furthermore, a majority of patients experience complex visual hallucinations that recur over time [7]. Another characteristic of DLB that is related to temporal aspects of brain function is a slowing of thinking and information processing referred to as bradyphrenia [8]. Overall, the transient nature of these symptoms suggests that functional rather than structural alterations might play a greater role in their aetiology [9].

The analysis of resting-state fMRI data allows study of changes in the functional architecture of the brain that are associated with neurodegenerative diseases. In particular, resting-state fMRI can be used to characterise large-scale functional networks, so-called resting-state networks (RSN), which are a set of spatially distinct brain regions that show coordinated activity in the absence of a specific task [10, 11]. Studying functional connectivity within and between these brain networks can shed light on spatial and temporal aspects of brain function in health and disease. Previous studies in DLB patients have reported changes in functional connectivity in networks associated with motor function, cognitive control, and attention [12–15]. However, whether these DLB-related changes occur early in the course of the disease remains an unanswered question. The first aim of this study was therefore to investigate changes in intra- and inter-network functional connectivity in MCI-LB patients compared to healthy controls and patients with MCI due to Alzheimer’s disease (MCI-AD).

More recently it has become evident that the assumption of temporal stationarity that underlies these traditional analyses of functional connectivity stands in contrast to the fact that connectivity can vary in both strength and directionality on a timescale of seconds to minutes [16, 17]. The focus has therefore shifted towards the analysis of dynamic or time-varying functional connectivity which aims to describe how connectivity changes over the duration of a scan [18, 19]. In a previous dynamic connectivity study we have reported that compared to controls, DLB patients spent more time in a state of low inter-network connectivity and showed difficulties in switching into more highly and specifically connected network configurations over time [20]. Furthermore, a relative reduction in the temporal variability of global network efficiency was observed in DLB patients indicating the presence of an abnormally rigid brain network [20, 21]. These results are remarkably similar to those that have been reported in Parkinson’s disease dementia (PDD) [22, 23] which shows large symptomatic and pathological overlap with DLB [24–26]. In the context of PDD, these changes are already evident at the MCI stage suggesting that they occur early in the course of the disease [22]. Therefore the second aim of this study was to test whether connectivity dynamics are also affected early in the context of DLB by investigating changes in time-varying functional connectivity in MCI-LB patients.

## Methods

### Participants

Recruitment and clinical assessment of participants for this study have been described previously [27]. Briefly, patients were recruited from local memory services and MCI was diagnosed by a consensus panel of three experienced old-age psychiatrists according to NIA-AA criteria [28]. Following a detailed clinical assessment, patients with a diagnosis of dementia or subjective cognitive impairment were excluded and all included patients had a CDR of 0 or 0.5. The panel rated the presence or absence of the core Lewy body symptoms (visual hallucinations, cognitive fluctuations, Parkinsonism, and REM sleep behaviour disorder) [2]. Additionally, participants had undergone dopaminergic imaging with ^123^I-N-fluoropropyl-2β-carbomethoxy-3β-(4-iodophenyl) single-photon emission computed tomography (FP-CIT SPECT) and ^123^iodine-metaiodobenzylguanidine (MIBG) myocardial scintigraphy.

A diagnosis of MCI due to Alzheimer’s disease (MCI-AD) was given to patients who had no core Lewy body symptoms, negative FP-CIT and MIBG findings, and evidence of cognitive decline that was characteristic of AD, i.e. they met the additional NIA-AA criterion for “aetiology of MCI consistent with AD pathophysiologic process” [28]. Probable MCI with Lewy bodies (MCI-LB) was diagnosed if a patient had two or more core Lewy body symptoms or one core symptom in addition to a positive FP-CIT and/or MIBG scan [4].

Of those participants who had undergone MR imaging, 38 participants were diagnosed with probable MCI-LB and 36 were diagnosed with MCI-AD. Healthy controls (*N* = 31) were recruited from friends and relatives of the patients and from a local research register and had no history of psychiatric or neurological illness and no evidence of cognitive decline.

Written informed consent was obtained from all participants prior to study participation and the study was approved by the local ethics committee.

### Data acquisition

MR imaging was performed on a 3 T Philips Intera Achieva scanner with an eight channel head coil receiver. Structural images were acquired with a magnetization prepared rapid gradient echo (MPRAGE) sequence, sagittal acquisition, echo time = 4.6 ms, repetition time (TR) = 8.3 ms, inversion time = 1250 ms, flip angle = 8°, SENSE factor = 2, in-plane resolution = 1.0 × 1.0 mm^2^, slice thickness = 1.0 mm. Resting-state scans were obtained with a gradient echo echo-planar imaging sequence with 33 contiguous axial slices, 290 volumes, anterior–posterior acquisition, in-plane resolution = 3.0 × 3.0 mm, slice thickness = 3.0 mm (1.0 mm gap), TR = 2072 ms, echo time = 30 ms, and field of view = 192 × 192 mm^2^. A fluid attenuated inversion recovery (FLAIR) sequence was acquired with repetition time = 11,000 ms, inversion time = 2800 ms, echo time = 25 ms voxel size = 0.94 × 0.94 mm, and 50 slices with thickness = 3.0 mm. Patients who were taking dopaminergic medication were scanned in the motor ON state.

### Preprocessing

Preprocessing of MRI data was performed using fMRIPrep version 20.0.6 [29].

The T1-weighted images were corrected for intensity non-uniformity and brain-extracted using the ANTs toolbox [30, 31]. Brain tissue segmentation into cerebrospinal fluid (CSF), grey matter (GM), and white matter (WM) was performed on the brain-extracted structural images using FAST in FSL (version 5.0.9) [32]. Volume-based spatial normalisation to standard space (MNI152NLin6Asym) was performed through non-linear registration with ANTs (version 2.2.0).

For each participant, a reference resting-state fMRI volume and its skull-stripped version were generated using a custom methodology of fMRIPrep. The reference image was then coregistered to the structural image using FSL’s FLIRT with boundary-based registration [33]. Head motion parameters with respect to the reference volume (transformation matrices and six corresponding rotation and translation parameters) were estimated before any spatiotemporal filtering using FSL’s MCFLIRT [33]. The resting-state fMRI images were then normalised to MNI space combining all spatial transformations (head motion correction, co-registration to structural images, and normalisation to MNI space) into a single step using Lanczos interpolation in ANTs. To estimate the extent of motion present in the fMRI data, framewise displacement (FD) was calculated [34] and participants with a mean FD above 0.5 mm were excluded from further analysis.

To further reduce the influence of motion, ICA-AROMA [35] was applied to the preprocessed fMRI data in MNI space after spatial smoothing with an isotropic 6 mm full-width at half maximum (FWHM) Gaussian kernel. Additionally, mean WM and CSF signals were estimated from the corresponding masks. These WM and CSF signals were then regressed out of the data together with a set of discrete cosine regressors to perform simultaneous band-pass filtering between 0.01 and 0.1 Hz. To avoid introducing previously removed noise signals back into the data, the whole regressor matrix was denoised with respect to the identified ICA-AROMA noise components using fsl_regfilt prior to nuisance regression [36]. Finally, grand-mean scaling was applied to the denoised resting-state fMRI data and data were resampled to a resolution of 4 mm^3^ using ANTs.

The brain masks estimated by fMRIPrep were combined across all participants to create a group brain mask by only including voxels that were non-zero in all subject-specific masks.

Areas of white matter hyperintensity (WMH) were identified from FLAIR images using in-house developed code in SPM [37] and total WMH volumes were estimated as a measure of vascular load.

### Analysis of static functional connectivity

**Fig. 1:**
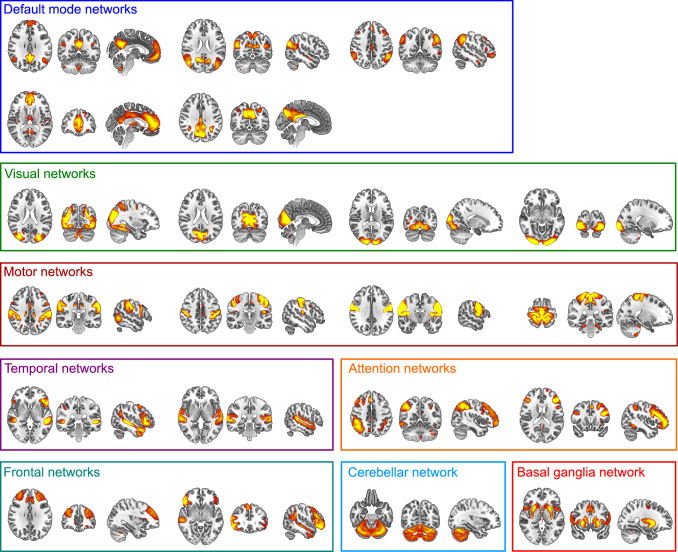
Included resting-state networks from the UK Biobank group-ICA (*d* = 21) overlaid on the MNI brain template. Spatial maps are thresholded at 5 < *z* < 15. Images are shown in radiological convention, i.e. the right side of the image corresponds to the left hemisphere

We used RSN templates from the UK Biobank study which were estimated by combining resting-state fMRI data from over 4000 UK Biobank participants and applying group independent component analysis (ICA) [38]. Group ICA decomposes the data into a specified number of networks and was run at two different dimensionalities (*d* = 25 and *d* = 100, referring to the number of distinct ICA components). Components that were classified as being of non-neuronal origin (e.g. due to head motion) were excluded from the analysis (four noise components for *d* = 25 and 45 noise components for *d* = 100). Furthermore, in the high-dimensional case four additional components were excluded because they were located mainly outside of the estimated group mask. Thus, the low-dimensional case included 21 RSNs (see Fig. 1) and the high-dimensional case included 51 RSNs (see Supplementary Figure S1) for further analysis.

Subsequently, subject-specific representations of the 21 (51) RSNs were estimated using group-information guided ICA (gig-ICA) [39] which results in subject-specific spatial maps and associated subject-specific time courses for each RSN.

Group differences in functional connectivity between controls, MCI-AD, and MCI-LB were assessed using Permutation Analysis of Linear Models (PALM) [40] with tail approximation [41] and the number of permutations set to 500. Threshold-free cluster enhancement (TFCE) was used for voxel-wise multiple comparison correction and p-values were also family-wise error corrected across the number of included RSNs (21 and 51, respectively) and the six group comparisons (two-sided tests for controls vs MCI-AD, controls vs MCI-LB, and MCI-AD vs MCI-LB) [42]. Covariates of no interest for age and sex were included in the model as well as a voxel-wise covariate for grey matter density [43].

The FSLnets package was used to assess between-network connectivity. Full and partial correlations were calculated between all pairs of RSNs and the correlation coefficients were converted to z-scores. PALM was used to assess group differences including covariates for age and sex. Results were FWE-corrected for multiple comparisons (across full and partial correlations, the number of RSN pairs, and the six different contrasts).

### Dynamic sliding-window analysis

Dynamic functional connectivity was assessed using a sliding-window analysis (see below, Allen et al., 2014). To remove potentially remaining noise sources, the subject-specific time courses resulting from gig-ICA were further processed in Matlab using functions from the Group ICA of fMRI toolbox (GIFT, http://mialab.mrn.org/software/gift/index.html). This postprocessing included detrending to remove linear, quadratic, and cubic trends, outlier detection using AFNI’s 3dDespike function, and interpolation of outliers using a third-order spline fit to the clean parts of the signal.

A tapered sliding window was created by convolving a rectangular window with a size of 22 TR (~ 44 s) with a Gaussian with sigma of 3 TR which was moved in steps of 1 TR. This resulted in a total of 269 overlapping windows. To assess the influence of the choice of window length on the results, the analysis was repeated using windows of length 18, 20, 24, and 26 TR.

A covariance matrix between all RSN pairs was estimated following the approach from [44]. Since estimation of covariance based on short time series can be noisy, the regularised inverse covariance matrix was estimated using the graphical LASSO approach. An L1-norm constraint was imposed on the inverse covariance matrix to achieve regularisation and promote sparsity. The L1 regularisation parameter *λ* was optimized for each participant individually by evaluating the log-likelihood of unseen time windows from the same participant using 20-fold cross-validation. All covariances were then converted to correlation values and transformed into *z*-scores using Fisher *r*-to-*z* transformation. To control for the effect of possible covariates the *z*-scores were then residualised with respect to age and sex using multiple linear regression [45].

The variability of functional connectivity over time was assessed by calculating the standard deviation of the correlation matrices across time windows. To assess whole-brain dynamics, the mean standard deviation across all RSN connections was calculated. Furthermore, the mean standard deviation for each network and for each connection were assessed separately.

To assess patterns of functional connectivity that reoccur over time, a k-means clustering analysis was applied to the windowed correlation matrices from all participants using the Manhattan distance function in Matlab. The clustering was repeated 500 times with random initialisation of cluster centroids to get a more stable solution. The optimal number of clusters *k* was determined based on the elbow criterion of the cluster validity index, i.e. the sum of within-cluster distances divided by the sum of between-cluster distances [44].

Group differences were assessed with respect to (1) frequency: proportion of windows assigned to each state, (2) mean dwell time: time spent in a state before transitioning to a different state, (3) number of transitions: overall number of state transitions, and (4) mean intertransition time: average time between two state transitions.

### Leading eigenvector dynamic analysis

In addition to the sliding-window analysis, we also tested an approach for the estimation of dynamic functional connectivity that does not rely on a sliding window, Leading Eigenvector Dynamic Analysis (LEiDA) [46]. The advantage of this approach is that it allows to estimate similarity between the time series of different brain regions instantaneously using the phase of signals (using the Hilbert transform).

For this analysis, 90 regions from the Automatic Anatomical Labelling (AAL) atlas were used [47]. These were then masked with the estimated group mask and atlas regions with less than 50% overlap with the group mask were excluded [48]. This led to the exclusion of 12 regions, thus including *N* = 78 regions from the AAL atlas for further analysis (see Supplementary Table S1).

Mean BOLD time series were extracted for each participant and each atlas region. To obtain a time-resolved connectivity matrix between all pairs of brain regions, the phase of the BOLD time series of each region was estimated using the Hilbert transform. The phase coherence between regions *n* and *p* at time *t* was then assessed using the following formula

$$\mathrm{dFC} \left(n,p,t\right)=\mathrm{cos}\left(\theta \left(n,t\right)-\theta \left(p,t\right)\right),$$

where $\theta$(n,t) is the phase of the BOLD signal in region n at time t. If two regions have temporarily aligned BOLD signals, i.e. they have similar phases, dFC will be close to 1. In contrast, if BOLD signals from two areas are orthogonal to each other, dFC will be close to 0. This results in a symmetric and square matrix for each timepoint with the number of rows and columns equal to the number of brain regions N in which the entry dFC(*n*,*p*,*t*) reflects the similarity between the BOLD signals of regions *n* and *p* at time *t*.

To study the evolution of dFC over time, the most common approach is to compare the *N* × *N* dFC(*t*) matrices obtained at each time point (see “Dynamic sliding-window analysis”). However, instead of considering all *N* × (*N* − 1)/2 distinct values of dFC, Cabral et al. [46] have shown that the leading eigenvector *V*_1_(*t*) at each time point can be used to capture the dominant connectivity pattern of dFC at time *t*. We therefore calculated a time-by-time matrix representing functional connectivity dynamics (FCD) where each entry FCD(*t*_1_,*t*_2_) corresponds to the cosine similarity between the leading eigenvectors of dFC at time *t*_1_ and *t*_2_, ranging from − 1 to 1.

To assess functional connectivity patterns that reoccur over time, a *k*-means clustering analysis was applied to the leading eigenvectors across all time points and participants using the Manhattan distance function in Matlab. Again, the clustering was repeated 500 times with random initialisation of cluster centroids and the optimal number of clusters *k* was determined using the elbow criterion. Group differences were assessed using the same metrics as for the sliding-window *k*-means analysis described in “Dynamic sliding-window analysis”.

### Statistical analysis

Statistical analyses were performed in SPSS and R (https://www.R-project.org/).

For the sliding-window analysis, the variability of functional connectivity of each RSN and each connection was compared between the groups using non-parametric multivariate ANOVAs (MANOVA, [49]) with diagnosis as the between-subject factor. For the LEiDA analysis, the mean similarity between time windows (from the FCD matrix) was compared between groups using a Kruskal–Wallis ANOVA.

The *k*-means measures from the sliding window and the LEiDA analysis (frequency and mean dwell time per state) were compared between the groups using non-parametric ANOVAs. The number of transitions and mean intertransition time were compared between the groups using univariate Kruskal–Wallis ANOVAs.

The effect of cholinesterase inhibitor use on dynamic connectivity measures in the MCI-LB patients was tested by comparing all dynamic connectivity measures between MCI-LB patients who were taking cholinesterase inhibitors (*N* = 14) to those MCI-LB patients not taking this medication (*N* = 16) using Mann–Whitney *U* tests. Furthermore, we repeated the analysis after excluding patients who were taking cholinesterase inhibitors, thus including 16 MCI-LB, 21 MCI-AD, and 24 healthy control participants.

As a supplementary analysis to assess whether vascular load might influence the functional connectivity results, we tested the inclusion of a covariate for WMH volume in the analyses. This did not change any of the results.

## Results

### Exclusion of participants

Two MCI-AD and one control participant were excluded due to restricted fields of view of the resting-state fMRI data. Additionally, six controls, five MCI-ADs, and seven MCI-LBs had to be excluded because of excessive motion. This resulted in 31 patients with MCI-LB, 28 patients with MCI-AD, and 24 controls for further analysis.

### Demographics

All three groups were similar in age and the two MCI groups were similar in terms of their overall cognitive impairment as measured by MMSE and ACE-R scores (see Table 1). The MCI-LB group included more male participants than the MCI-AD and the healthy control groups. The MCI-LB group had higher cognitive fluctuation and visual hallucination scores and more MCI-LB patients were taking cholinesterase inhibitors compared to the MCI-AD group. No participants were taking antipsychotic drugs. Seven MCI-AD, 12 MCI-LB, and two control participants were taking anti-depressants. Additionally, one MCI-AD, eight MCI-LB, and two control participants were taking hypnotic/anxiolytic medication.

**Table 1:** Demographic and clinical variables, mean (standard deviation)

	HC (*N* = 24)	MCI-AD (*N* = 28)	MCI-LB (*N* = 31)	Group differences
Male:female	17:7	14:14	29:2	*χ*^2^ = 14.0, *p* = 0.001^a^ *p*(HC,MCI-AD) = 0.13 *p*(HC,MCI-LB) = 0.02 *p*(MCI-AD,MCI-LB) < 0.001
Age	73.5 (7.6)	76.2 (7.9)	74.7 (6.6)	*F*(2,80) = 0.9, *p* = 0.41^b^
AChEI	–	5 (19%)^e^	14 (47%)^f^	*χ*^2^ = 5.1, *p* = 0.08^c^
PD meds	–	0^e^	2 (7%)^f^	*χ*^2^ = 2.2, *p* = 0.32^c^
Years of education	14.5 (3.7)^g^	12.9 (3.5)	12.1 (3.0)	*F*(2,77) = 3.4, *p* = 0.04^b^ *p*(HC,MCI-AD) = 0.32 *p*(HC,MCI-LB) = 0.03 *p*(MCI-AD,MCI-LB) = 1.0
ACE-R	92.3 (4.4)	82.2 (8.8)	83.4 (9.5)	*t*_57_ = 0.5, *p* = 0.62^d^
MMSE	28.3 (1.1)	27.0 (2.2)	26.6 (2.6)	*t*_57_ = 0.6, *p* = 0.61^d^
UPDRS III	5.3 (4.3)	16.2 (15.1)	22.8 (14.9)	*t*_57_ = 1.7, *p* = 0.1^d^
DCFS	–	6.7 (1.9)^h^	8.8 (3.5)^i^	*t*_49_ = 2.6, *p* = 0.01^d^
CAF total	–	1.3 (2.4)^h^	4.2 (4.5)^i^	*t*_49_ = 2.8, *p* = 0.008^d^
NPI total	–	7.6 (8.3)^h^	14.9 (12.6)^i^	*t*_49_ = 2.4, *p* = 0.02^d^
NEVHI	–	0.6 (1.2)^j^	3.3 (4.4)	*t*_56_ = 3.1, *p* = 0.003^d^
Mean FD (mm)	0.25 (0.09)	0.28 (0.10)	0.26 (0.11)	*F*(2,80) = 0.6, *p* = 0.59^b^
Max FD (mm)	0.94 (0.66)	1.3 (0.65)	1.1 (0.73)	*F*(2,80) = 1.7, *p* = 0.19^b^

*ACE-R* Addenbrooke’s Cognitive Examination—Revised, *AChEI* number of patients taking acetylcholinesterase inhibitors, *CAF total* Clinician Assessment of Fluctuation total score, *DCFS* Dementia cognitive fluctuation scale, *FD* framewise displacement, *HC* healthy controls, *MCI-AD* mild cognitive impairment with Alzheimer’s disease, *MCI-LB* probable mild cognitive impairment with Lewy bodies, *MMSE* Mini Mental State Examination, *NEVHI* North-East Visual Hallucinations Interview, *NPI* Neuropsychiatric Inventory, *PD meds* number of patients taking dopaminergic medication for the management of Parkinson’s disease symptoms, *UPDRS III* Unified Parkinson’s Disease Rating Scale III (motor subsection)

^a^Chi-square test HC, MCI-AD, MCI-LB; ^b^Univariate ANOVA HC, MCI-AD, MCI-LB; ^c^Chi-square test MCI-AD, MCI-LB; ^d^Student’s *t* test MCI-AD, MCI-LB, ^e^*N* = 26, ^f^*N* = 30, ^g^*N* = 22, ^h^*N* = 23, ^i^*N* = 28, ^j^*N* = 27

There was no difference in mean or maximum FD between the three groups (see Table 1). There was no difference in total WMH volume (normalised with respect to total brain volume) between the groups (*F*(2,80) = 1.8, *p* = 0.17).

### Static functional connectivity

PALM did not identify any significant differences in the group comparison of within- and between-network connectivity for any RSN, for the low- (*d* = 21) and the high-dimensional (*d* = 51) Biobank RSNs.

### Dynamic sliding-window analysis

**Fig. 2:**
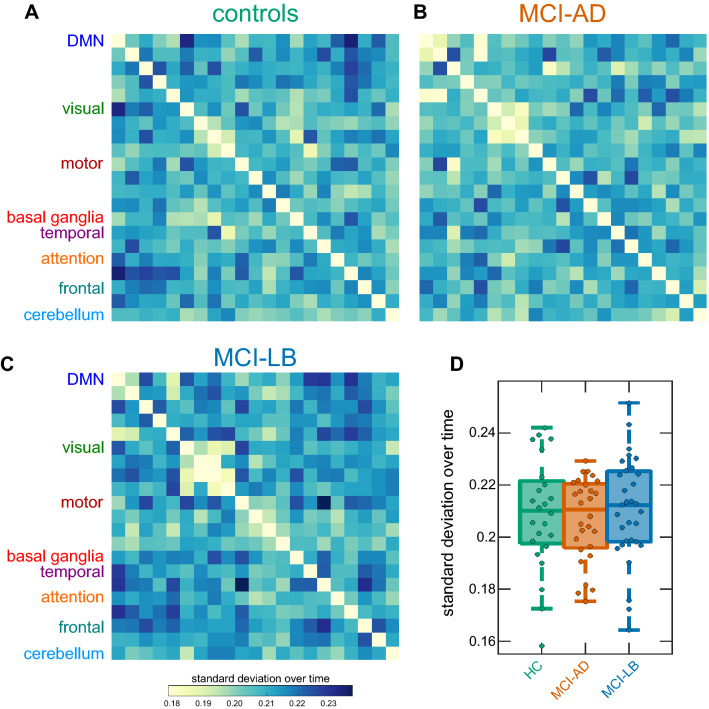
Results from sliding-window dynamic functional connectivity analysis. **a**–**c** Matrices represent mean standard deviation over time for the HC, MCI-AD, and MCI-LB groups. **d** Boxplot of group comparison of mean standard deviation across all connections. In the boxplot the central line corresponds to the sample median, the upper and lower border of the box represent the 25th and 75th percentile, respectively, and the length of the whiskers is 1.5 times the interquartile range. *DMN* default mode network, *HC* healthy controls, *MCI-AD* mild cognitive impairment with Alzheimer’s disease, *MCI-LB* mild cognitive impairment with Lewy bodies

Figure 2a–c shows matrices representing the mean standard deviation of the strengths of each RSN-to-RSN connection within each group. The mean variability of RSN connectivity, across all connections, was not significantly different between groups (*H*_2_ = 0.55, *p* = 0.76, Fig. 2d). When considering average variability for each RSN separately, the overall MANOVA did not show a significant effect of diagnosis (*F*(4,155) = 0.61, *p* = 0.65). Similarly, when considering each individual RSN-to-RSN connection, the MANOVA did not reveal a significant group effect (*F*(32,1267) = 0.93, *p* = 0.57). These results were consistent across different window sizes (Supplementary Figure S2 and Supplementary Table S2) and when using the higher-dimensional (*d* = 51) group-ICA components (Supplementary Figure S3 and Supplementary Table S4).

**Fig. 3:**
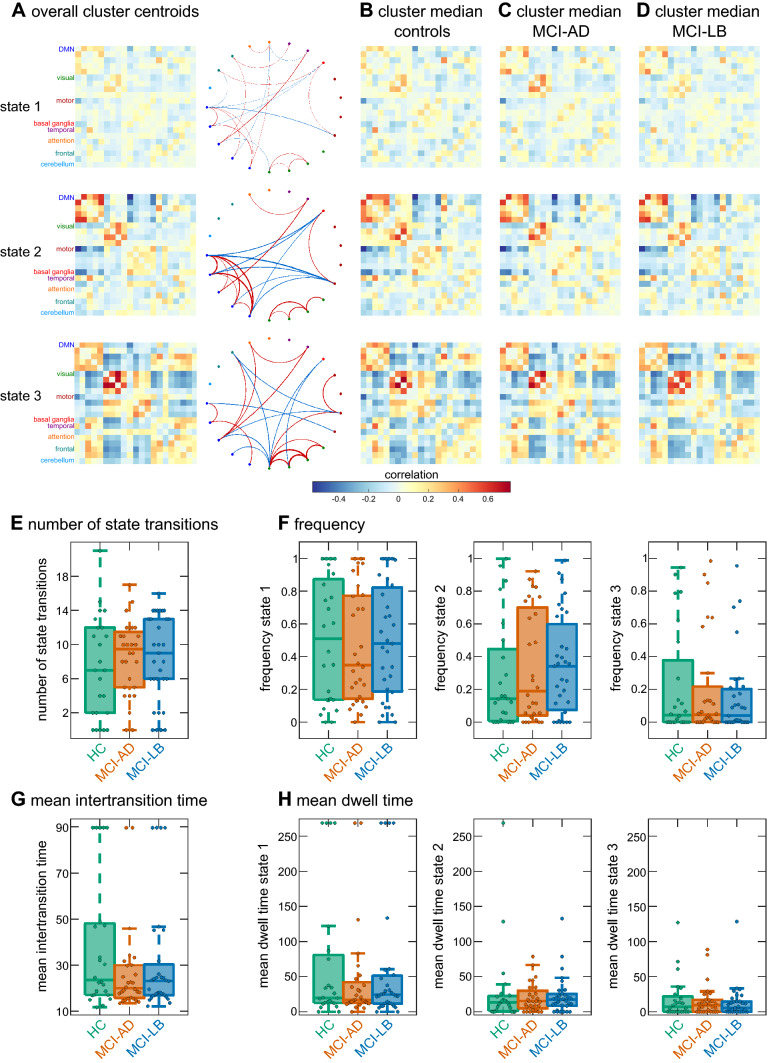
Results from the sliding-window *k*-means analysis with low-dimensional Biobank RSNs (*d* = 21). **a** Centroids resulting from clustering on all windows and participants. The network graphs are showing only the 5% strongest positive (red) and negative (blue) connections. **b** Cluster medians in the healthy control group. **c** Cluster medians in the MCI-AD group. **d** Cluster medians in the MCI-LB group. **e** Group comparison of the overall number of state transitions. **f** Group comparison of frequency of occurrence of the three states. **g** Group comparison of mean time between two state transitions. **h** Comparison of mean dwell time of the three states. In the boxplots the central line corresponds to the sample median, the upper and lower border of the box represent the 25th and 75th percentile, respectively, and the length of the whiskers is 1.5 times the interquartile range. *HC* healthy controls, *MCI-AD* mild cognitive impairment with Alzheimer’s disease, *MCI-LB* mild cognitive impairment with Lewy bodies

An optimal number of *k* = 3 clusters was determined for the clustering analysis using the elbow criterion (Supplementary Figure S4). State 1 was characterised by overall sparse connectivity with slightly stronger connectivity between different parts of the default mode network and between the different visual networks (Fig. 3a–d). In contrast, state 2 was characterised by stronger overall connectivity, especially within default mode and visual networks, and strong negative connectivity between the default mode and motor and basal ganglia networks. State 3 showed particularly strong connectivity within visual networks and strong negative connectivity between the visual and the other networks. State 1 was the most common state, accounting for 49% of all time windows across all participants, whereas 32% of time windows were assigned to state 2 and participants spent 19% of their time in state 3.

There were no group differences between controls, MCI-AD, and MCI-LB in terms of the number of state transitions (*H*_2_ = 0.99, *p* = 0.61, Fig. 3e), the mean intertransition time (*H*_2_ = 0.86, *p* = 0.65, Fig. 3g), the frequency of the three states (*F*(4,170) = 0.21, *p* = 0.94, Fig. 3f) or mean dwell time per state (*F*(4,174) = 0.17, *p* = 0.96, Fig. 3h). These results remained consistent when varying the number of clusters *k* from 2 to 6 (Supplementary Figure S5 and Supplementary Table S6), when repeating the analysis with different window sizes (Supplementary Figure S6 and Supplementary Table S6), and when using the higher-dimensional group-ICA components (Supplementary Figures S7-S9 and Supplementary Table S8).

### Leading eigenvector dynamic analysis

An optimal number of *k* = 3 clusters was determined for the LEiDA clustering analysis (Supplementary Figure S4). The overall similarity between time windows did not differ between groups (*H*_2_ = 3.4, *p* = 0.18).

**Fig. 4:**
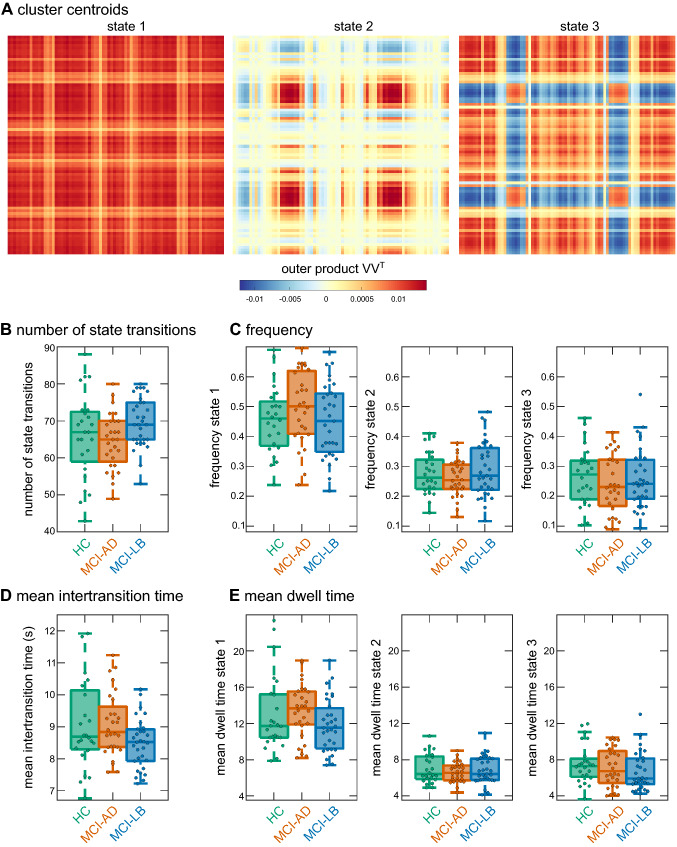
Results from the LEiDA *k*-means analysis. **a** Centroids resulting from clustering on all windows and participants, i.e. the outer product of the vector V that represents each cluster centroid. **b** Group comparison of the overall number of state transitions. **c** Group comparison of frequency of occurrence of the three states. **d** Group comparison of mean time between two state transitions. **e** Comparison of mean dwell time of the three states. In the boxplots the central line corresponds to the sample median, the upper and lower border of the box represent the 25th and 75th percentile, respectively, and the length of the whiskers is 1.5 times the interquartile range*.* *HC* healthy controls, *MCI-AD* mild cognitive impairment with Alzheimer’s disease, *MCI-LB* mild cognitive impairment with Lewy bodies

Figure 4a shows the cluster centroids for the three states. Each cluster centroid is a vector V and the outer product VV^T^ represents a N x N connectivity pattern which indicates the contribution of each brain area to that pattern. State 1 was the most common state accounting for 47% of all time points across participants. It corresponds to a state of global BOLD coherence, i.e. the BOLD signals of all brain areas exhibit a strong coherence (Fig. 4). State 2, which accounted for 28% of time points, shows strong coherence only between different occipital regions (across both hemispheres). State 3 was present in 25% of time points and exhibited overall strong coherence between brain regions, except for occipital areas that appear to be decoupled from other brain regions.

There were no group differences between controls, MCI-AD, and MCI-LB in terms of the number of state transitions (*H*_2_ = 5.6, *p* = 0.06, Fig. 4b), the mean intertransition time (*H*_2_ = 4.4, *p* = 0.11, Fig. 4c), the frequency of the three states (*F*(4,146) = 0.8, *p* = 0.51, Fig. 4b) or mean dwell time per state (*F*(6,217) = 1.1, *p* = 0.34, Fig. 4d). When repeating the analysis with *k* = 2, a significant difference in the number of state transitions (*H*_2_ = 9.3, *p* = 0.01) and the mean intertransition time (*H*_2_ = 9.2, *p* = 0.01) was found. Post-hoc tests revealed that the number of state transitions was significantly higher and the mean intertransition time significantly lower in MCI-LB compared to MCI-AD (Supplementary Table S10). However, these results were not reproducible for higher values of k.

### Effect of cholinesterase inhibitor use

None of the dynamic connectivity measures (from sliding window and LEiDA analyses) showed any differences between MCI-LB patients who were taking cholinesterase inhibitors and those MCI-LB patients not taking this medication (all *p* > 0.1). Repeating the analysis after excluding patients who were taking cholinesterase inhibitors did not change any of the results (see Supplementary Tables S3, S5, S7, S9, and S11).

## Discussion

In this work we investigated resting-state functional connectivity in patients with MCI-LB compared to healthy controls and MCI-AD patients from different perspectives. We did not find any significant changes in the MCI-LB group compared to controls and no differences between the two MCI groups, using both static as well as dynamic connectivity measures. To ensure that results were not biased by choosing a specific dimensionality for the RSN estimation or by the particular method used for dynamic analysis, we repeated the analysis using low- and high-dimensional RSN templates and two different strategies to assess connectivity dynamics. Additionally, whenever an analysis involved assigning a specific value to a parameter (i.e. window size, number of clusters) we repeated the analyses using a range of parameter values.

In the dynamic *k*-means analysis, we found recurring connectivity patterns that are comparable to previous studies, i.e. one state of overall low inter-network connectivity that accounts for a majority of the time windows and one or more states that are characterised by a stronger and more specific connectivity profile and account for a smaller number of time windows [20, 22, 50–52]. Previous studies at the dementia stage in both DLB and PDD patients have shown that patients tend to spend more time in a state of low overall connectivity and show difficulties to switch into states of higher connectivity [20, 22]. Importantly, it has been shown that in the context of Parkinson’s disease these changes occur early and can already be observed in PD-MCI patients [22, 51]. In contrast, we did not observe early changes in the frequency or mean dwell time of connectivity states in our MCI-LB group. It is possible that this difference is influenced by the fact that PD-MCI patients already have a longer disease duration compared to MCI-LB patients with comparable levels of cognitive impairment. One could also argue that PD-MCI may represent a more pure alpha-synucleinopathy whereas in MCI-LB there may be more Alzheimer’s disease co-pathology which could influence the phenotype [53, 54].

Sliding-window methods have been criticised for requiring the choice of a window size which affects their temporal resolution and statistical validity [55, 56]. The second dynamic connectivity method that we applied, LEiDA, overcomes this issue using the phase of the signal to obtain instantaneous measures of dynamic connectivity [46]. Again, we found states that are comparable to previous studies: The most prevalent state was a state of global BOLD coherence whereas the other less frequently occurring states were characterised by more specific coherence patterns [46]. However, similar to the sliding-window analysis, this method did not detect any significant changes in the MCI-LB group compared to healthy controls or MCI-AD patients.

In previous studies of the same patient cohort we have found changes in MCI-LB patients compared to controls and MCI-AD patients not only with respect to their cognitive profile [57, 58], but also in terms of resting-state EEG measures [27] as well as structural changes within the nucleus basalis of Meynert [59]. This indicates that changes in brain function are already evident in these patients despite their early stage of disease. The lack of differences found in the present study therefore suggests that the fMRI analyses conducted here might not be sensitive enough to discern early and subtle changes in MCI-LB patients. From the absence of significant findings, however, we cannot simply conclude that there are no differences in functional connectivity between the groups as the absence of evidence does not automatically provide evidence for the absence of an effect. A particular challenge with studying MCI groups is their large heterogeneity which influences our ability to detect differences at the group level. There might be subtle changes in functional connectivity in MCI-LB patients that will only become detectable later on in the course of the disease. These analyses should therefore be repeated in independent cohorts, ideally taking advantage of longitudinal fMRI data to study changes that occur over the disease course.

This work has some limitations. First, some of our MCI patients were taking cholinesterase inhibitors and dopaminergic medication which might have normalised their fMRI characteristics [60–62]. However, when comparing those MCI-LB patients who were taking cholinesterase inhibitors to those who were not, we did not find any significant differences for any of the dynamic connectivity measures. Furthermore, restricting the analysis to those participants who were not taking cholinesterase inhibitors did not change the findings. The prescription rates for cholinesterase inhibitors in this cohort reflect local use for treatment of neuropsychiatric symptoms in Lewy body disease and are in line with recent guidelines [63]. Only two of the MCI-LB patients were taking dopaminergic medication which precludes any further analysis of its effect on functional connectivity metrics. Another potential limitation is the sex imbalance between the three groups which reflects differences in prevalence of AD and DLB in men and women [64, 65]. We have included a covariate for sex in all analyses; however, this imbalance still remains as a limitation of this work.

## Conclusion

In summary, we did not find any evidence for significant early changes in static or dynamic functional connectivity in MCI-LB patients compared to controls and MCI-AD. While MCI-LB patients already show clear functional abnormalities on EEG measures, the fMRI analyses presented here do not appear to be sensitive enough to detect such early differences in brain function between the groups.

## Supplementary Information

Below is the link to the electronic supplementary material.

Supplementary file1 (DOCX 5686 KB)

## Data Availability

The code to reproduce the analyses presented in this manuscript is available from the corresponding author.

## Declarations

### Availability of data and material

The data that support the findings of this study are available from the corresponding author, upon reasonable request.
