# Supplementary material for: Functional connectivity in mild cognitive impairment with Lewy bodies
Source: J Neurol. 2021 Apr 29;268(12):4707–20. doi: 10.1007/s00415-021-10580-z (PMC8563567; doi:10.1007/s00415-021-10580-z)
Supplement: Supplementary file 1 — Supplementary file1 (DOCX 5686 KB) [file 415_2021_10580_MOESM1_ESM.docx]

Supplementary Material

Functional connectivity in mild cognitive impairment with Lewy bodies

Julia Schumacher, John-Paul Taylor, Calum A. Hamilton, Michael Firbank, Paul C. Donaghy, Gemma Roberts, Louise Allan, Rory Durcan, Nicola Barnett, John T. O’Brien, Alan J. Thomas

Correspondence: [julia.schumacher@newcastle.ac.uk](mailto:julia.schumacher@newcastle.ac.uk)


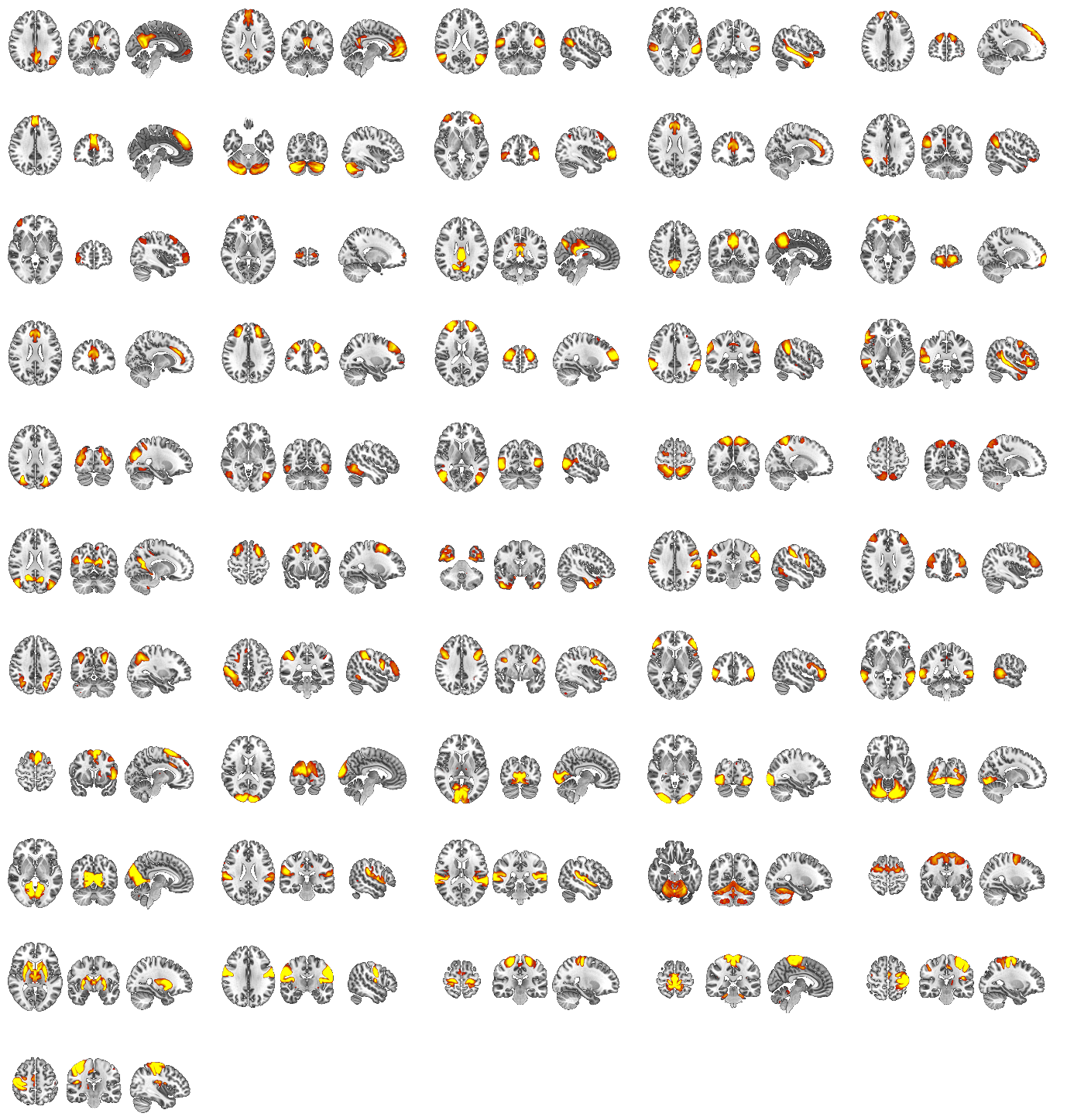


Supplementary Figure S1: Included resting state networks from the high-dimensional UK Biobank group-ICA (d=51) overlaid on the MNI brain template. Spatial maps are thresholded at 5 <z<15. Images are shown in radiological convention, i.e. the right side of the image corresponds to the left hemisphere.

Supplementary Table S1: Included regions from the AAL atlas.

| Region name | |
| --- | --- |
| Precentral L | Precentral R |
| Frontal Sup L | Frontal Sup R |
| Frontal Mid L | Frontal Mid R |
| Frontal Inf Oper L | Frontal Inf Oper R |
| Frontal Inf Tri L | Frontal Inf Tri R |
| Frontal Inf Orb L | Frontal Inf Orb R |
| Rolandic Oper L | Rolandic Oper R |
| Supp Motor Area L | Supp Motor Area R |
| Olfactory L | Olfactory R |
| Frontal Sup Medial L | Frontal Sup Medial R |
| Insula L | Insula R |
| Cingulum Ant L | Cingulum Ant R |
| Cingulum Mid L | Cingulum Mid R |
| Cingulum Post L | Cingulum Post R |
| Hippocampus L | Hippocampus R |
| ParaHippocampal L | ParaHippocampal R |
| Amygdala L | Amygdala R |
| Calcarine L | Calcarine R |
| Cuneus L | Cuneus R |
| Lingual L | Lingual R |
| Occipital Sup L | Occipital Sup R |
| Occipital Mid L | Occipital Mid R |
| Occipital Inf L | Occipital Inf R |
| Fusiform L | Fusiform R |
| Postcentral L | Postcentral R |
| Parietal Sup L | Parietal Sup R |
| Parietal Inf L | Parietal Inf R |
| SupraMarginal L | SupraMarginal R |
| Angular L | Angular R |
| Precuneus L | Precuneus R |
| Paracentral Lobule L | Paracentral Lobule R |
| Caudate L | Caudate R |
| Putamen L | Putamen R |
| Pallidum L | Pallidum R |
| Thalamus L | Thalamus R |
| Heschl L | Heschl R |
| Temporal Sup L | Temporal Sup R |
| Temporal Pole Sup L | Temporal Pole Sup R |
| Temporal Mid L | Temporal Mid R |


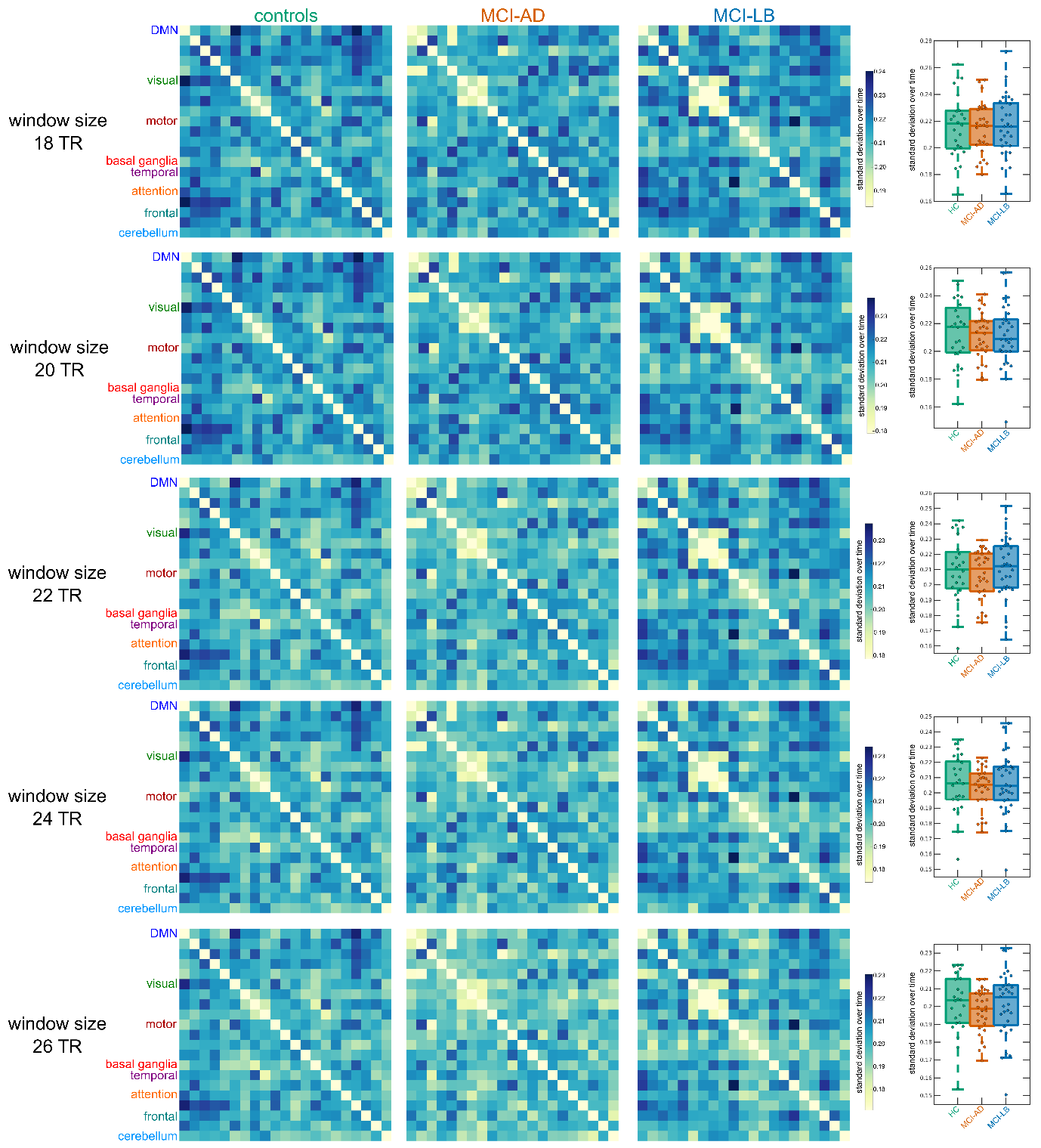


Supplementary Figure S2: Mean standard deviation matrices and group comparison of mean standard deviation across all connections between healthy controls, MCI-AD, and MCI-LB for different window sizes ranging from 18 TR to 26 TR, using the low-dimensional (d=21) Biobank RSNs. See Supplementary Table S3 for results from statistical tests.

HC, healthy controls; MCI-AD, mild cognitive impairment with Alzheimer’s disease; MCI-LB, mild cognitive impairment with Lewy bodies; TR, repetition time

Supplementary Table S2: Group comparison of variability of connectivity between HC, MCI-AD, and MCI-LB from sliding-window analysis with low-dimensional Biobank RSNs (d=21).

| Measure | window size | test | group comparison |
| --- | --- | --- | --- |
| mean variability of connectivity (across all connections) | | | |
|  | 18 TR | Kruskal-Wallis ANOVA | H_2_=0.12, p=0.94 |
|  | 20 TR | Kruskal-Wallis ANOVA | H_2_=0.21, p=0.90 |
|  | 24 TR | Kruskal-Wallis ANOVA | H_2_=0.38, p=0.83 |
|  | 26 TR | Kruskal-Wallis ANOVA | H_2_=2.1, p=0.36 |
| variability of connectivity for each RSN separately | | | |
|  | 18 TR | non-parametric MANOVA | F(3,124)=0.43, p=0.74 |
|  | 20 TR | non-parametric MANOVA | F(3,137)=0.5, p=0.71 |
|  | 24 TR | non-parametric MANOVA | F(5,184)=0.70, p=0.62 |
|  | 26 TR | non-parametric MANOVA | F(5,213)=1.15, p=0.33 |
| variability of connectivity for each connection separately | | | |
|  | 18 TR | non-parametric MANOVA | F(21,829)=0.84, p=0.67 |
|  | 20 TR | non-parametric MANOVA | F(26,1035)=0.90, p=0.61 |
|  | 24 TR | non-parametric MANOVA | F(42,1667)=0.95, p=0.57 |
|  | 26 TR | non-parametric MANOVA | F(82,2051)=1.03, p=0.42 |

Supplementary Table S3: Group comparison of variability of connectivity between HC, MCI-AD, and MCI-LB from sliding-window analysis with low-dimensional Biobank RSNs (d=21), restricting the analysis to participants who were not taking cholinesterase inhibitors (21 MCI-AD, 16 MCI-LB, 24 controls).

| Measure | window size | test | group comparison |
| --- | --- | --- | --- |
| mean variability of connectivity (across all connections) | | | |
|  | 18 TR | Kruskal-Wallis ANOVA | H_2_=0.16, p=0.93 |
|  | 20 TR | Kruskal-Wallis ANOVA | H_2_=0.55, p=0.76 |
|  | 22 TR | Kruskal-Wallis ANOVA | H_2_=0.35, p=0.84 |
|  | 24 TR | Kruskal-Wallis ANOVA | H_2_=0.77, p=0.68 |
|  | 26 TR | Kruskal-Wallis ANOVA | H_2_=2.5, p=0.29 |
| variability of connectivity for each RSN separately | | | |
|  | 18 TR | non-parametric MANOVA | F(3,88)=0.40, p=0.76 |
|  | 20 TR | non-parametric MANOVA | F(3,96)=0.55, p=0.67 |
|  | 22 TR | non-parametric MANOVA | F(4,107)=0.55, p=0.69 |
|  | 24 TR | non-parametric MANOVA | F(4,123)=0.67, p=0.62 |
|  | 26 TR | non-parametric MANOVA | F(5,139)=1.20, p=0.32 |
| variability of connectivity for each connection separately | | | |
|  | 18 TR | non-parametric MANOVA | F(20,552)=0.79, p=0.73 |
|  | 20 TR | non-parametric MANOVA | F(22,614)=0.84, p=0.67 |
|  | 22 TR | non-parametric MANOVA | F(27,772)=0.85, p=0.68 |
|  | 24 TR | non-parametric MANOVA | F(33,932)=0.88, p=0.67 |
|  | 26 TR | non-parametric MANOVA | F(40,1127)=1.0, p=0.47 |


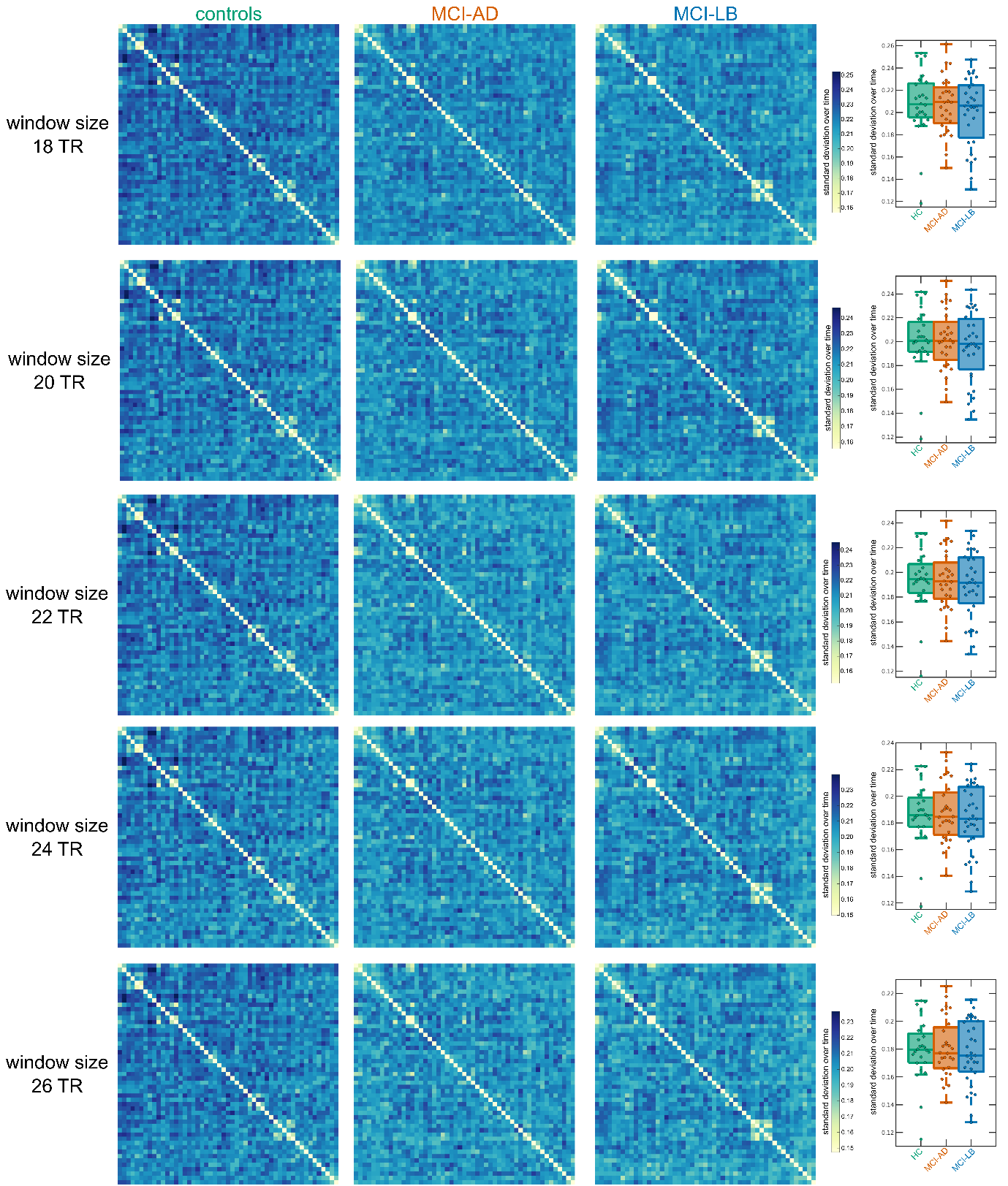


Supplementary Figure S3: Mean standard deviation matrices and group comparison of mean standard deviation across all connections between healthy controls, MCI-AD, and MCI-LB for different window sizes ranging from 18 TR to 26 TR, using the high-dimensional (d=51) Biobank RSNs. See Supplementary Table S4 for results from statistical tests.

HC, healthy controls; MCI-AD, mild cognitive impairment with Alzheimer’s disease; MCI-LB, mild cognitive impairment with Lewy bodies; TR, repetition time

Supplementary Table S4: Group comparison of variability of connectivity between HC, MCI-AD, and MCI-LB from sliding-window analysis with high-dimensional Biobank RSNs (d=51).

| Measure | window size | test | group comparison |
| --- | --- | --- | --- |
| mean variability of connectivity (across all connections) | | | |
|  | 18 | Kruskal-Wallis ANOVA | H_2_=0.75, p=0.69 |
|  | 20 | Kruskal-Wallis ANOVA | H_2_=0.45, p=0.80 |
|  | 22 | Kruskal-Wallis ANOVA | H_2_=1.2, p=0.56 |
|  | 24 | Kruskal-Wallis ANOVA | H_2_=0.45, p=0.80 |
|  | 26 | Kruskal-Wallis ANOVA | H_2_=1.4, p=0.49 |
| variability of connectivity for each RSN separately | | | |
|  | 18 | non-parametric MANOVA | F(3,119)=0.60, p=0.62 |
|  | 20 | non-parametric MANOVA | F(3,122)=0.4, p=0.76 |
|  | 22 | non-parametric MANOVA | F(3,136)=0.88, p=0.47 |
|  | 24 | non-parametric MANOVA | F(4,142)=0.51, p=0.71 |
|  | 26 | non-parametric MANOVA | F(4,166)=0.87, p=0.49 |
| variability of connectivity for each connection separately | | | |
|  | 18 | non-parametric MANOVA | F(20,779)=0.86, p=0.64 |
|  | 20 | non-parametric MANOVA | F(22,881)=0.78, p=0.76 |
|  | 22 | non-parametric MANOVA | F(30,1177)=0.93, p=0.58 |
|  | 24 | non-parametric MANOVA | F(33,1318)=0.85, p=0.71 |
|  | 26 | non-parametric MANOVA | F(43,1692)=0.92, p=0.61 |

Supplementary Table S5: Group comparison of variability of connectivity between HC, MCI-AD, and MCI-LB from sliding-window analysis with high-dimensional Biobank RSNs (d=51), restricting the analysis to participants who were not taking cholinesterase inhibitors (21 MCI-AD, 16 MCI-LB, 24 controls).

| Measure | window size | test | group comparison |
| --- | --- | --- | --- |
| mean variability of connectivity (across all connections) | | | |
|  | 18 | Kruskal-Wallis ANOVA | H_2_=1.1, p=0.59 |
|  | 20 | Kruskal-Wallis ANOVA | H_2_=0.63, p=0.73 |
|  | 22 | Kruskal-Wallis ANOVA | H_2_=1.7, p=0.42 |
|  | 24 | Kruskal-Wallis ANOVA | H_2_=0.82, p=0.67 |
|  | 26 | Kruskal-Wallis ANOVA | H_2_=2.3, p=0.32 |
| variability of connectivity for each RSN separately | | | |
|  | 18 | non-parametric MANOVA | F(3,81)=0.62, p=0.63 |
|  | 20 | non-parametric MANOVA | F(3,84)=0.41, p=0.74 |
|  | 22 | non-parametric MANOVA | F(3,91)=0.87, p=0.33 |
|  | 24 | non-parametric MANOVA | F(3,96)=0.57, p=0.66 |
|  | 26 | non-parametric MANOVA | F(4,111)=1.1, p=0.48 |
| variability of connectivity for each connection separately | | | |
|  | 18 | non-parametric MANOVA | F(17,466)=0.83, p=0.63 |
|  | 20 | non-parametric MANOVA | F(20,555)=0.74, p=0.87 |
|  | 22 | non-parametric MANOVA | F(24,677)=0.89, p=0.62 |
|  | 24 | non-parametric MANOVA | F(27,762)=0.83, p=0.72 |
|  | 26 | non-parametric MANOVA | F(34,959)=0.93, p=0.59 |


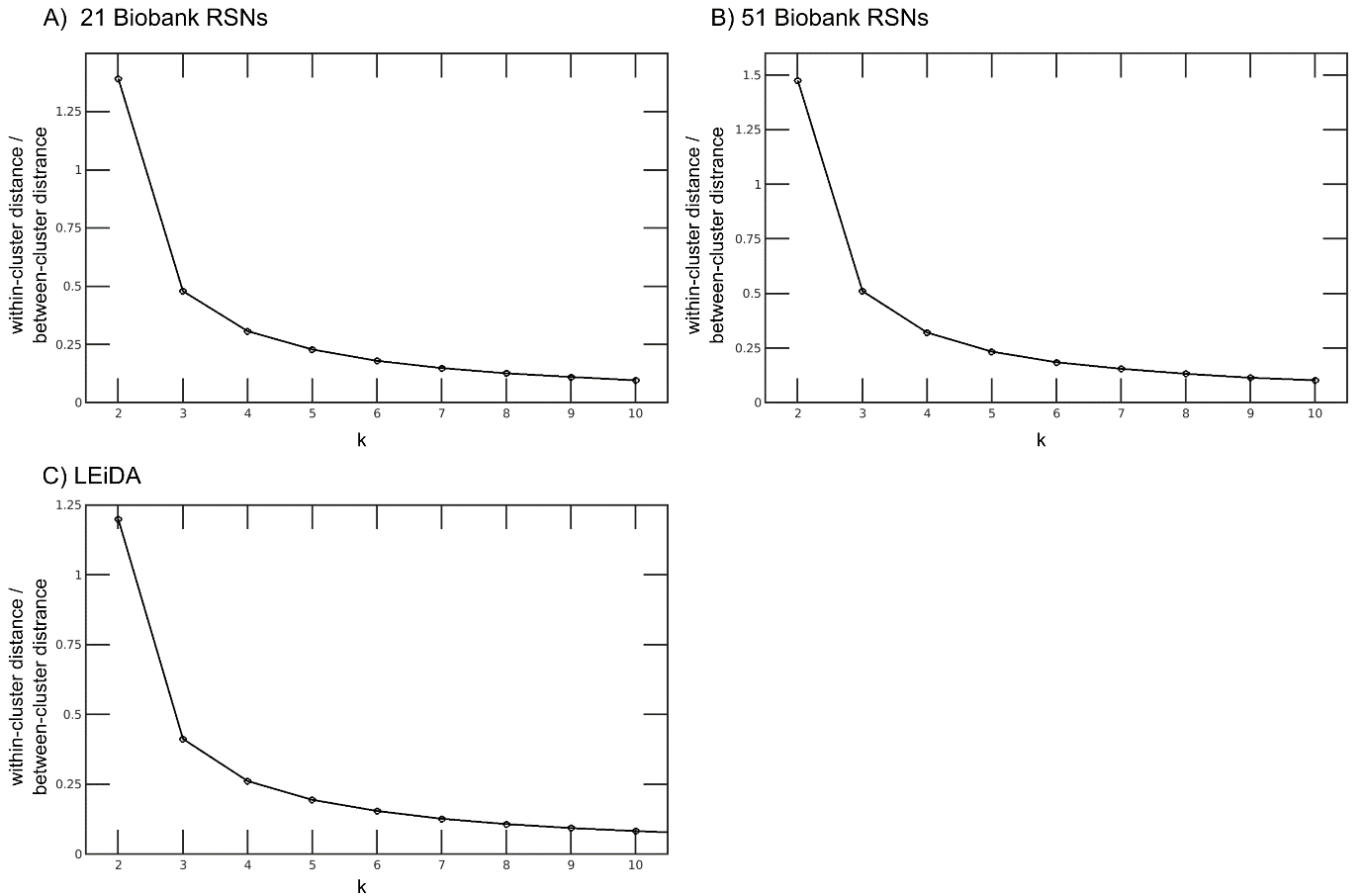


Supplementary Figure S4: Elbow plot of the ratio of within-cluster distance to between-cluster distance for A) clustering analysis with sliding-window dynamic connectivity matrices from 21 Biobank RSNs, B) clustering analysis with sliding-window dynamic connectivity matrices from 51 Biobank RSNs, and C) clustering analysis with leading eigenvectors from Leading Eigenvector Dynamic Analysis (LEiDA).


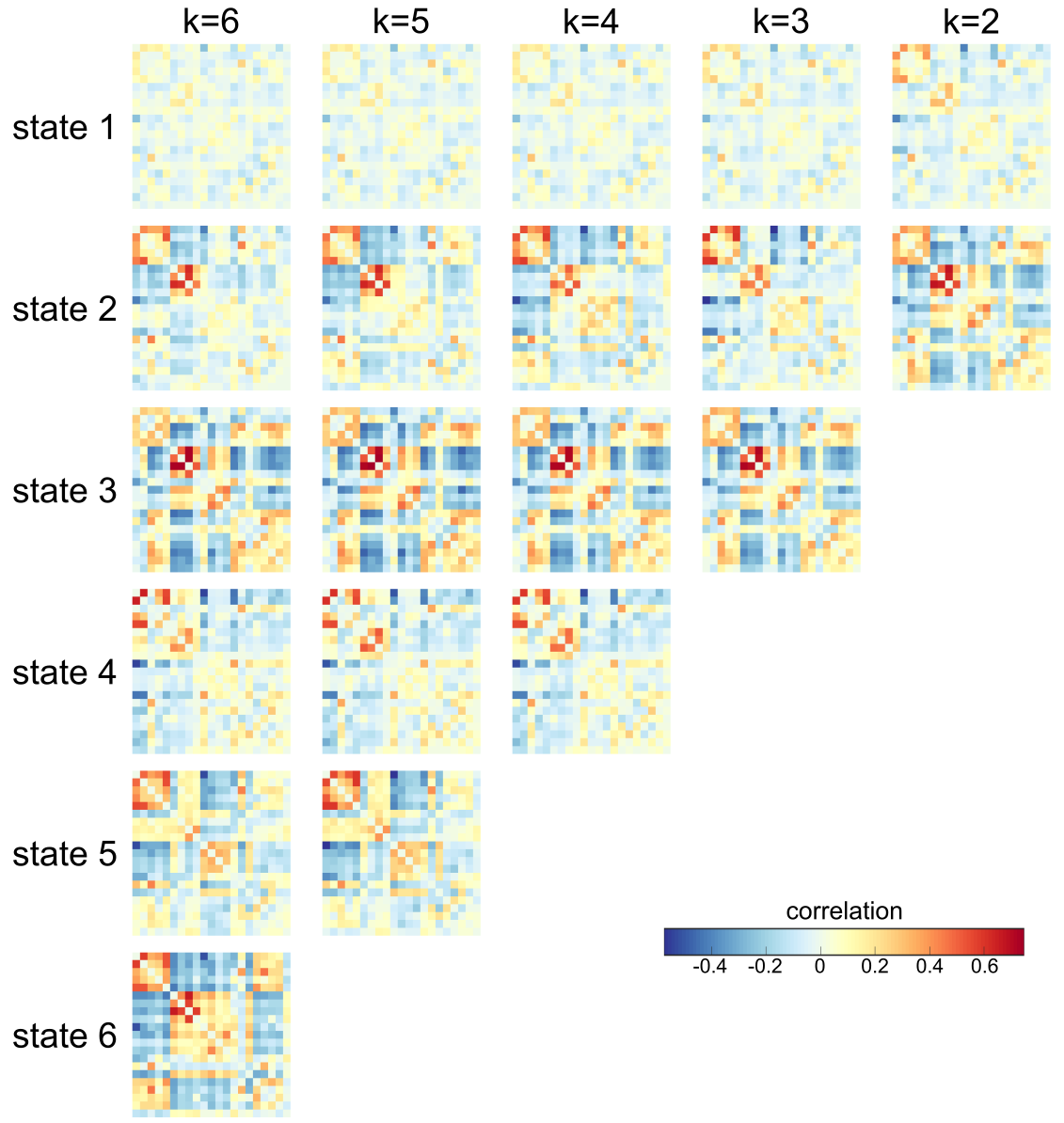


Supplementary Figure S5: Cluster centroids from sliding-window k-means analysis for different values of k, using the low-dimensional Biobank RSNs (d=21). Results of group comparisons of the different k-means measures can be found in Supplementary Table S5.


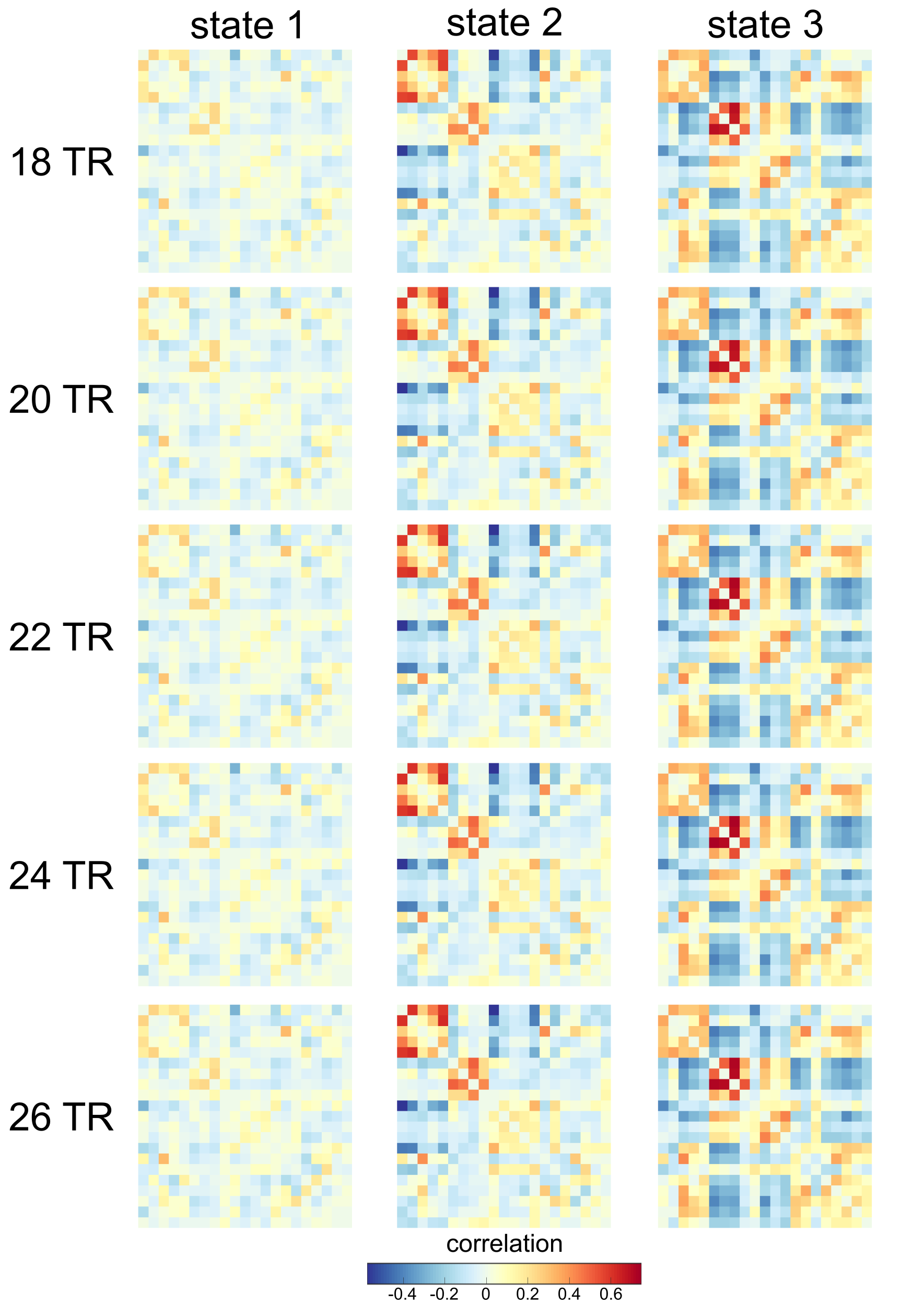


Supplementary Figure S6: Cluster centroids from sliding-window k-means analysis with k=3 and different window sizes, using the low-dimensional Biobank RSNs (d=21). Results of group comparisons of the different k-means measures can be found in Supplementary Table S5.

TR, repetition time

Supplementary Table S6: Group comparison of k-means measures between HC, MCI-AD, and MCI-LB from sliding-window analysis with low-dimensional Biobank RSNs (d=21) for different values of k and different window sizes.

|  | | number of transitions | intertransition time | frequency | mean dwell time |
| --- | --- | --- | --- | --- | --- |
| k=2 | | H_2_=1.96, p=0.38 | H_2_=2.16, p=0.34 | F(2,79)=0.19, p=0.83 | F(2,86)=0.17, p=0.87 |
| k=3 | |  |  |  |  |
|  | 18 TR | H_2_=2.07, p=0.36 | H_2_=2.83, p=0.24 | F(4,169)=0.27, p=0.91 | F(4,172)=0.43, p=0.81 |
|  | 20 TR | H_2_=1.25, p=0.54 | H_2_=1.49, p=0.48 | F(4,170)=0.28, p=0.90 | F(4,175)=0.27, p=0.91 |
|  | 24 TR | H_2_=0.43, p=0.81 | H_2_=0.37, p=0.83 | F(4,170)=0.24, p=0.93 | F(4,172)=0.19, p=0.95 |
|  | 26 TR | H_2_=0.83, p=0.66 | H_2_=0.38, p=0.83 | F(4,171)=0.21, p=0.94 | F(4,177)=0.22, p=0.94 |
| k=4 | | H_2_=1.62, p=0.45 | H_2_=1.55, p=0.46 | F(6,236)=0.52, p=0.79 | F(6,241)=0.65, p=0.70 |
| k=5 | | H_2_=0.62, p=0.73 | H_2_=0.81, p=0.67 | F(8,304)=0.46, p=0.88 | F(8,317)=0.52, p=0.85 |
| k=6 | | H_2_=1.63, p=0.44 | H_2_=0.13, p=0.94 | F(9,366)=0.47, p=0.90 | F(10,378)=0.35, p=0.97 |

Supplementary Table S7: Group comparison of k-means measures between HC, MCI-AD, and MCI-LB from sliding-window analysis with low-dimensional Biobank RSNs (d=21) for different values of k and different window sizes, restricting the analysis to participants who were not taking cholinesterase inhibitors (21 MCI-AD, 16 MCI-LB, 24 controls).

|  | | number of transitions | intertransition time | frequency | mean dwell time |
| --- | --- | --- | --- | --- | --- |
| k=2 | | H_2_=0.96, p=0.62 | H_2_=1.2, p=0.56 | F(2,56)=0.48, p=0.56 | F(2,61)=0.35, p=0.71 |
| k=3 | |  |  |  |  |
|  | 18 TR | H_2_=1.2, p=0.55 | H_2_=1.5, p=0.47 | F(4,121)=0.30, p=0.90 | F(5,127)=0.57, p=0.71 |
|  | 20 TR | H_2_=0.59, p=0.74 | H_2_=0.98, p=0.61 | F(4,120)=0.37, p=0.87 | F(4,119)=0.32, p=0.88 |
|  | 22 TR | H_2_=0.34, p=0.84 | H_2_=0.63, p=0.73 | F(4,120)=0.21, p=0.94 | F(4,121)=0.16, p=0.97 |
|  | 24 TR | H_2_=0.33, p=0.85 | H_2_=0.79, p=0.67 | F(4,117)=0.25, p=0.90 | F(4,119)=0.25, p=0.92 |
|  | 26 TR | H_2_=0.49, p=0.78 | H_2_=0.74, p=0.69 | F(4,118)=0.15, p=1.0 | F(4,121)=0.21, p=0.94 |
| k=4 | | H_2_=0.58, p=0.75 | H_2_=1.1, p=0.59 | F(6,156)=0.28, p=0.93 | F(6,156)=0.36, p=0.96 |
| k=5 | | H_2_=0.03, p=0.99 | H_2_=0.82, p=0.66 | F(7,204)=0.24, p=0.98 | F(8,317)=0.52, p=0.85 |
| k=6 | | H_2_=0.03, p=0.98 | H_2_=0.31, p=0.86 | F(9,248)=0.31, p=0.99 | F(10,378)=0.35, p=0.97 |


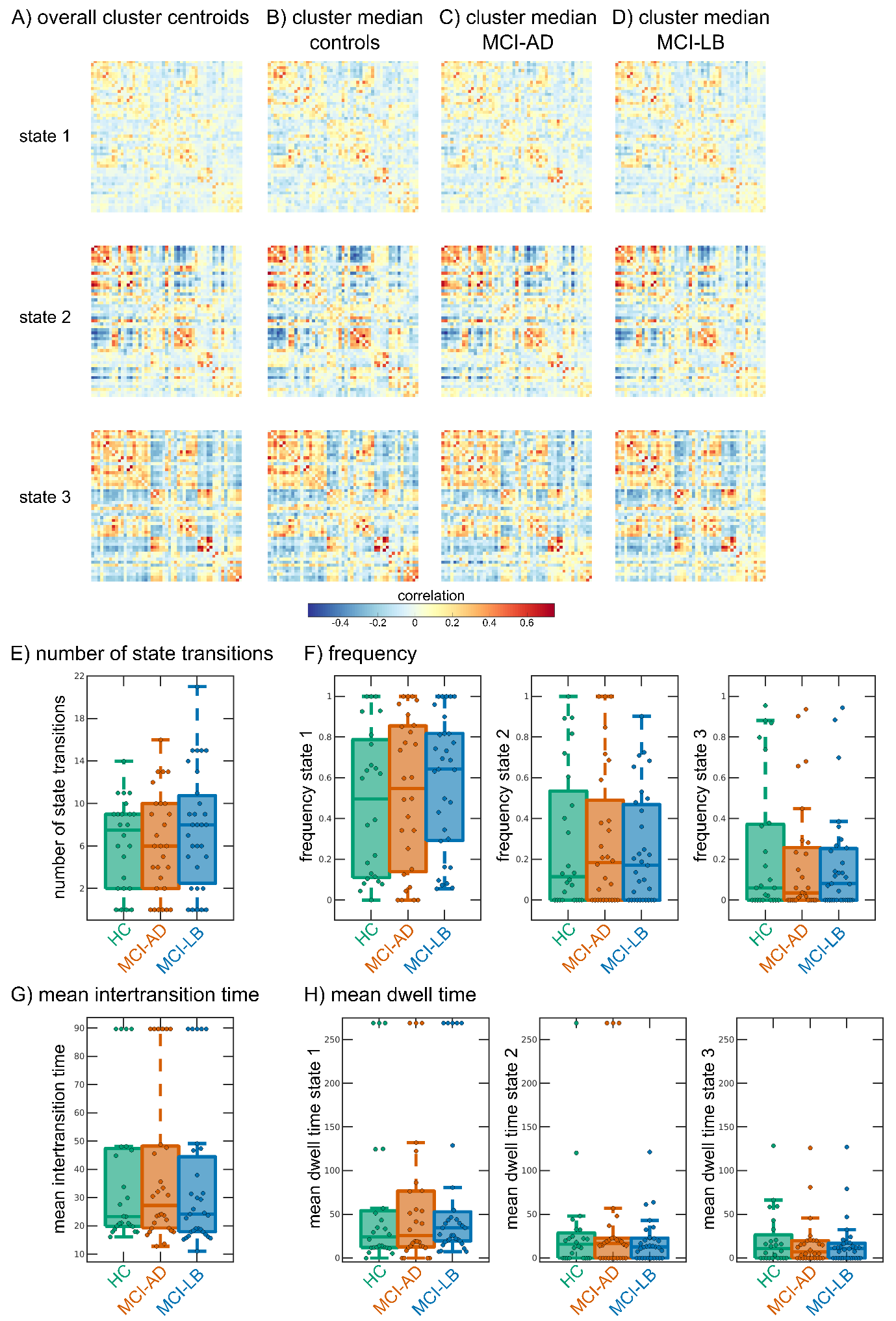


Supplementary Figure S7: Figure 1: Results from the sliding window k-means analysis with high-dimensional Biobank RSNs (d=51). A) Centroids resulting from clustering on all windows and participants. B) Cluster medians in the healthy control group. C) Cluster medians in the MCI-AD group. D) Cluster medians in the MCI-LB group. E) Group comparison of the overall number of state transitions. F) Group comparison of frequency of occurrence of the three states. G) Group comparison of mean time between two state transitions. H) Comparison of mean dwell time of the three states. Results of statistical tests for the different k-means measures can be found in Supplementary Table S6.

In the boxplot the central line corresponds to the sample median, the upper and lower border of the box represent the 25th and 75th percentile, respectively, and the length of the whiskers is 1.5 times the interquartile range.

HC, healthy controls; MCI-AD, mild cognitive impairment with Alzheimer’s disease; MCI-LB, mild cognitive impairment with Lewy bodies


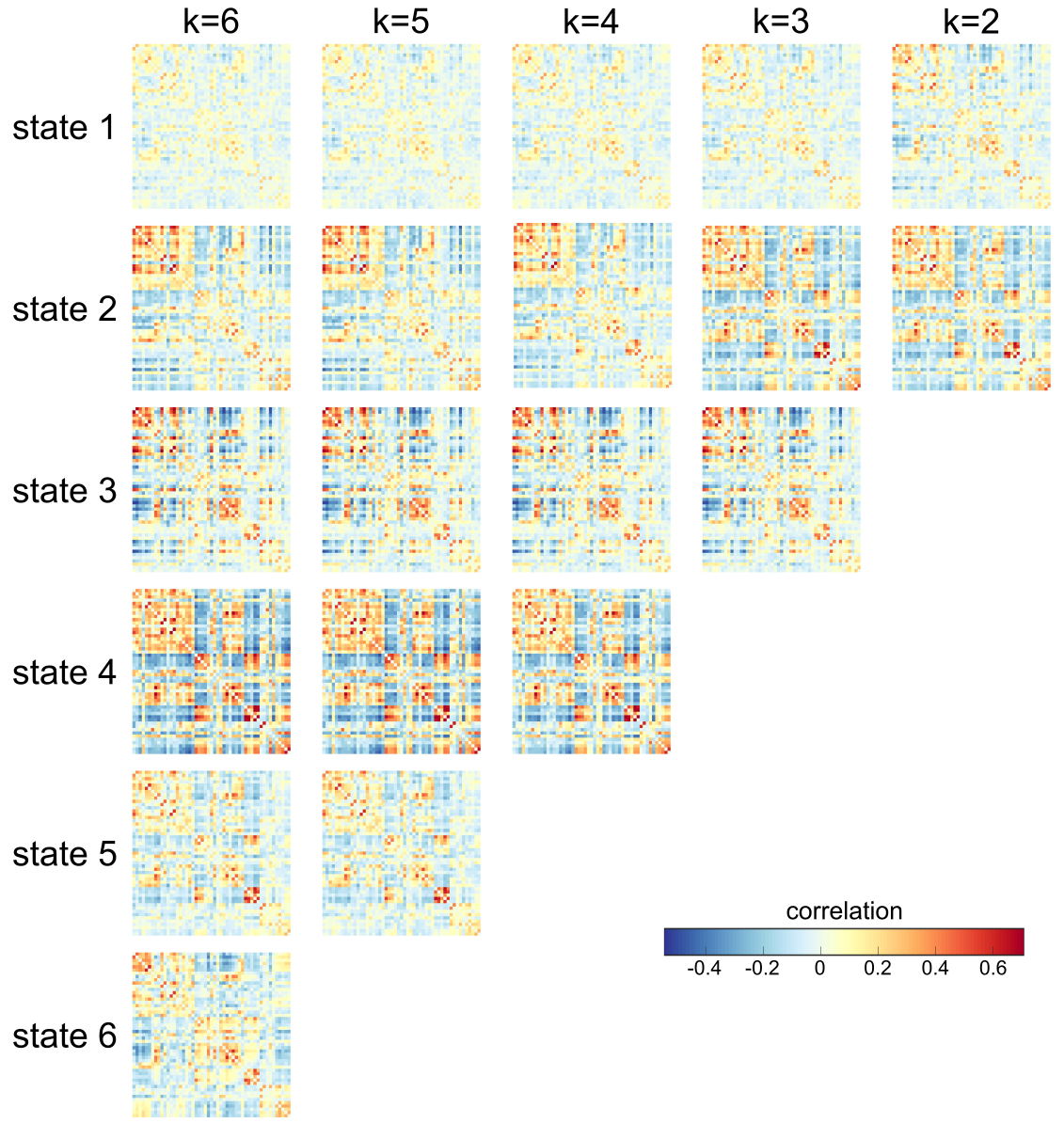


Supplementary Figure S8: Cluster centroids from sliding-window k-means analysis for different values of k, using the high-dimensional Biobank RSNs (d=51). Results of group comparisons of the different k-means measures can be found in Supplementary Table S6.


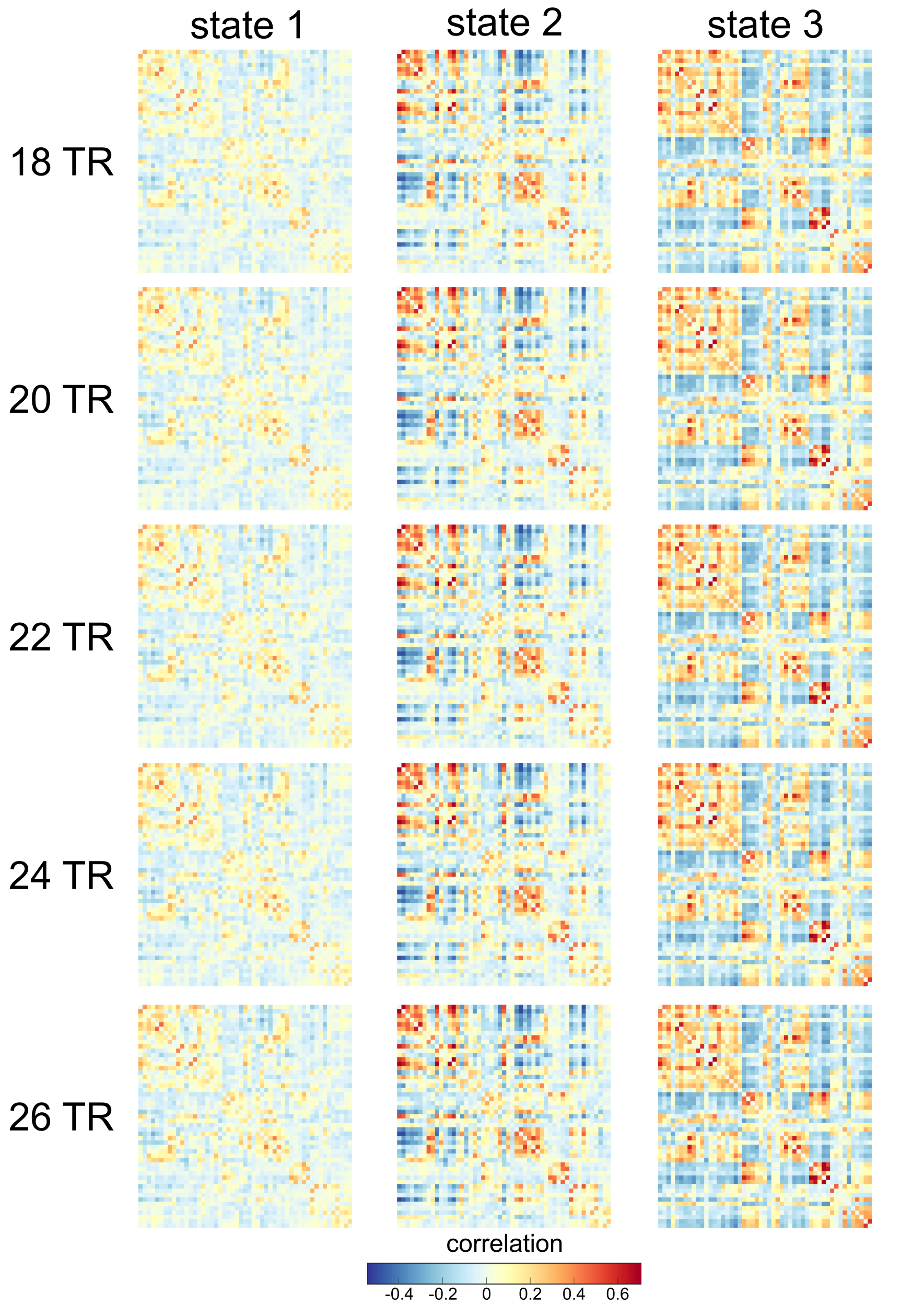


Supplementary Figure S9: Cluster centroids from sliding-window k-means analysis with k=3 and different window sizes, using the high-dimensional Biobank RSNs (d=51). Results of group comparisons of the different k-means measures can be found in Supplementary Table S6.

TR, repetition time Supplementary Table S8: Group comparison of k-means measures between HC, MCI-AD, and MCI-LB from sliding-window analysis with high-dimensional Biobank RSNs (d=51) for different values of k and different window sizes.

|  | | number of transitions | intertransition time | frequency | mean dwell time |
| --- | --- | --- | --- | --- | --- |
| k=2 | | H_2_=1.32, p=0.52 | H_2_=1.39, p=0.50 | F(2,79)=0.01, p=0.99 | F(2,86)=0..07, p=0.95 |
| k=3 | |  |  |  |  |
|  | 18 TR | H_2_=0.50, p=0.78 | H_2_=0.50, p=0.7 | F(4,167)=0.17, p=0.96 | F(4,174)=0.20, p=0.95 |
|  | 20 TR | H_2_=0.20, p=0.91 | H_2_=0.18, p=0.92 | F(4,168)=0.16, p=0.96 | F(4,172)=0.27, p=0.91 |
|  | 22 TR | H_2_=0.75, p=0.69 | H_2_=0.89, p=0.64 | F(4,168)=0.20, p=0.94 | F(4,175)=0.32, p=0.89 |
|  | 24 TR | H_2_=0.46, p=0.80 | H_2_=0.41, p=0.81 | F(4,168)=0.24, p=0.92 | F(4,174)=0.41, p=0.82 |
|  | 26 TR | H_2_=0.64, p=0.73 | H_2_=1.10, p=0.59 | F(4,167)=0.30, p=0.89 | F(4,173)=0.37, p=0.84 |
| k=4 | | H_2_=1.95, p=0.38 | H_2_=1.93, p=0.38 | F(6,240)=0.31, p=0.93 | F(6,244)=0.28, p=0.95 |
| k=5 | | H_2_=0.31, p=0.86 | H_2_=0.006, p=1.0 | F(8,306)=0.27, p=0.97 | F(8,308)=0.37, p=0.93 |
| k=6 | | H_2_=0.16, p=0.92 | H_2_=0.41, p=0.82 | F(10,385)=0.27, p=0.99 | F(10,387)=0.33, p=0.97 |

Supplementary Table S9: Group comparison of k-means measures between HC, MCI-AD, and MCI-LB from sliding-window analysis with high-dimensional Biobank RSNs (d=51) for different values of k and different window sizes, restricting the analysis to participants who were not taking cholinesterase inhibitors (21 MCI-AD, 16 MCI-LB, 24 controls).

|  | | number of transitions | intertransition time | frequency | mean dwell time |
| --- | --- | --- | --- | --- | --- |
| k=2 | | H_2_=1.0, p=0.60 | H_2_=0.86, p=0.65 | F(2,56)=0.51, p=0.66 | F(2,62)=0.62, p=0.56 |
| k=3 | |  |  |  |  |
|  | 18 TR | H_2_=0.44, p=0.80 | H_2_=0.34, p=0.84 | F(4,121)=0.24, p=0.93 | F(4,124)=0.37, p=0.85 |
|  | 20 TR | H_2_=0.46, p=0.79 | H_2_=0.48, p=0.79 | F(4,121)=0.25, p=0.92 | F(4,123)=0.60, p=0.68 |
|  | 22 TR | H_2_=0.13, p=0.94 | H_2_=0.25, p=0.88 | F(4,121)=0.30, p=0.89 | F(5,127)=0.62, p=0.67 |
|  | 24 TR | H_2_=0.16, p=0.92 | H_2_=0.48, p=0.79 | F(4,122)=0.39, p=0.83 | F(5,127)=0.62, p=0.67 |
|  | 26 TR | H_2_=0.66, p=0.72 | H_2_=1.1, p=0.58 | F(4,122)=0.49, p=0.76 | F(4,126)=0.64, p=0.65 |
| k=4 | | H_2_=0.54, p=0.76 | H_2_=0.38, p=0.83 | F(6,168)=0.11, p=1.0 | F(6,166)=0.30, p=0.93 |
| k=5 | | H_2_=0.21, p=0.90 | H_2_=0.53, p=0.77 | F(7,204)=0.15, p=1.0 | F(8,308)=0.37, p=0.93 |
| k=6 | | H_2_=0.25, p=0.88 | H_2_=0.98, p=0.61 | F(9,260)=0.31, p=0.98 | F(10,387)=0.33, p=0.97 |


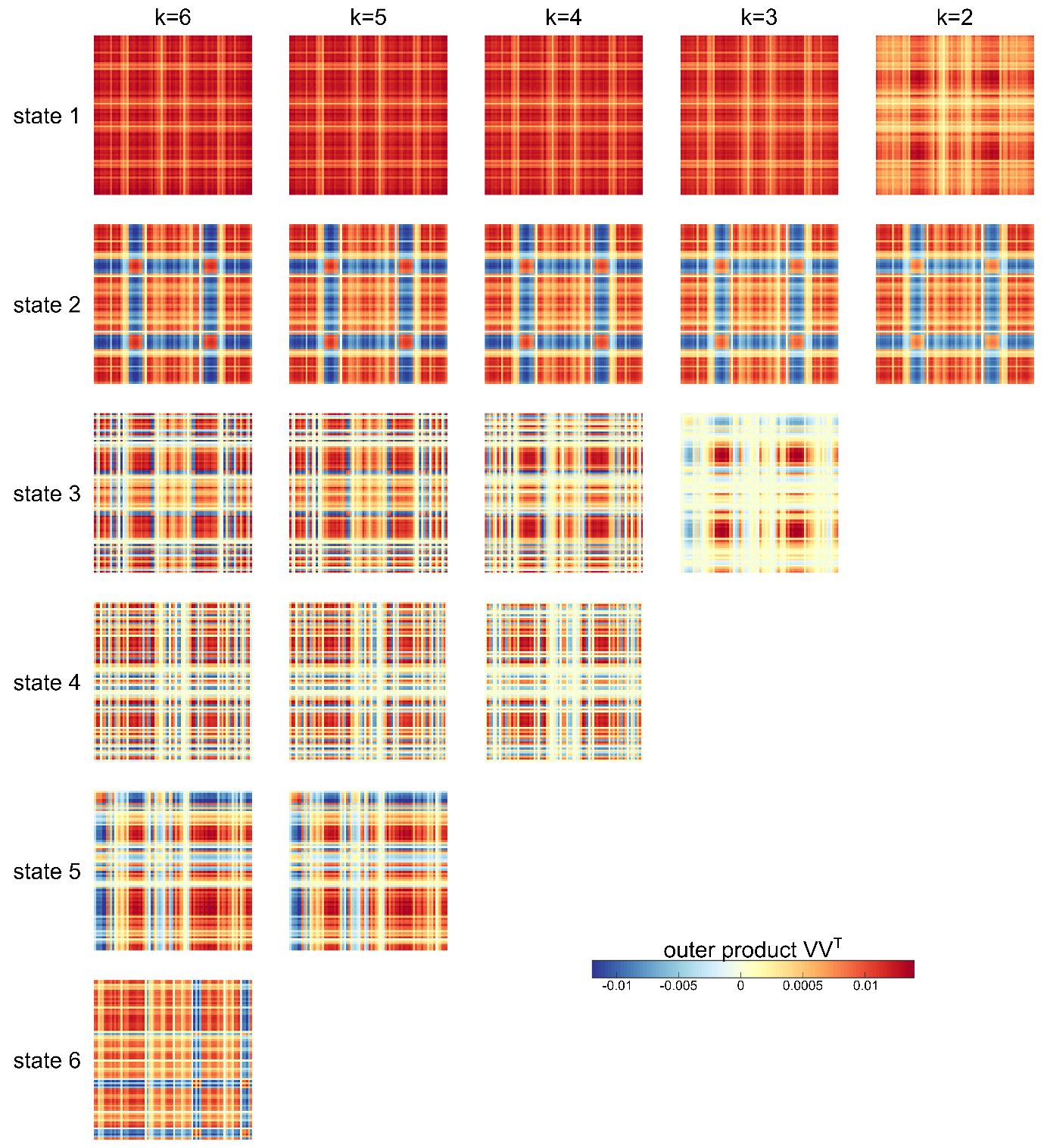


Figure S10: Cluster centroids from LEiDA k-means analysis for different values of k. Results of group comparisons of the different k-means measures can be found in Supplementary Table S7.

Supplementary Table S10: Group comparison of k-means measures between HC, MCI-AD, and MCI-LB from Leading Eigenvector Dynamic Analysis (LEiDA) for different values of k.

|  | number of transitions | intertransition time | frequency | mean dwell time |
| --- | --- | --- | --- | --- |
| k=2 | H_2_=9.3, p=0.01 | H_2_=9.2, p=0.01 | F(2,79)=0.5, p=0.60 | F(3,125)=1.7, p=0.16 |
|  | p(HC, MCI-AD)=0.40  p(HC, MCI-LB)=0.50  p(MCI-AD, MCI-LB)=0.007 | p(HC, MCI-AD)=0.54  p(HC, MCI-LB)=0.38  p(MCI-AD, MCI-LB)=0.007 | F(4,146)=0.8, p=0.51 | F(6,217)=1.1, p=0.34 |
| k=4 | H_2_=5.4, p=0.07 | H_2_=4.7, p=0.10 | F(6,228)=0.8, p=0.59 | F(7,289)=0.5, p=0.81 |
| k=5 | H_2_=0.5, p=0.76 | H_2_=1.0, p=0.59 | F(8,299)=1.2, p=0.29 | F(9,349)=1.2, p=0.31 |
| k=6 | H_2_=1.5, p=0.48 | H_2_=1.9, p=0.39 | F(10,380)=1.0, p=0.43 | F(11,432)=1.0, p=0.45 |

Supplementary Table S11: Group comparison of k-means measures between HC, MCI-AD, and MCI-LB from Leading Eigenvector Dynamic Analysis (LEiDA) for different values of k, restricting the analysis to participants who were not taking cholinesterase inhibitors (21 MCI-AD, 16 MCI-LB, 24 controls).

|  | number of transitions | intertransition time | frequency | mean dwell time |
| --- | --- | --- | --- | --- |
| k=2 | H_2_=3.7, p=0.16 | H_2_=3.5, p=0.17 | F(2,56)=0.48, p=0.62 | F(3,91)=1.2, p=0.30 |
| k=3 | H_2_=4.2, p=0.13 | H_2_=3.9, p=0.14 | F(4,107)=0.59, p=0.67 | F(5,152)=1.2, p=0.30 |
| k=4 | H_2_=3.2, p=0.20 | H_2_=2.9, p=0.24 | F(6,160)=0.73, p=0.62 | F(7,199)=0.82, p=0.57 |
| k=5 | H_2_=0.9, p=0.63 | H_2_=2.0, p=0.36 | F(8,213)=0.95, p=0.47 | F(9,246)=1.1, p=0.38 |
| k=6 | H_2_=1.7, p=0.43 | H_2_=2.6, p=0.27 | F(9,264)=0.74, p=0.68 | F(10,292)=1.0, p=0.42 |
